# Case Report: COVID-19 with Bilateral Adrenal Hemorrhage

**DOI:** 10.4269/ajtmh.20-0722

**Published:** 2020-07-17

**Authors:** Jorge Álvarez-Troncoso, Miriam Zapatero Larrauri, M. Dolores Montero Vega, Rebeca Gil Vallano, Esmeralda Palmier Peláez, Patricia Martín Rojas-Marcos, Fátima Martín-Luengo, Paula Lázaro del Campo, Carmen Rosario Herrero Gil, Elena Trigo Esteban

**Affiliations:** 1 Internal Medicine Department, La Paz University Hospital, Madrid, Spain;; 2 Endocrinology Department, La Paz University Hospital, Madrid, Spain;; 3 Microbiology Department, Head of the Serology, Molecular Biology Laboratory, La Paz University Hospital, Madrid, Spain;; 4 Radiology Department, La Paz University Hospital, Madrid, Spain;; 5 Universidad Autónoma de Madrid, Madrid, Spain;; 6 Hematology Department, La Paz University Hospital, Madrid, Spain;; 7 High Level Isolation Unit, Tropical and Travel Medicine National Referral Unit, Clinical COVID-19 Team, La Paz University Hospital, Madrid, Spain

## Abstract

A 70-year-old Dominican Republic man presented with lower back pain for 10 days. Fifteen days before pain onset, he had low-grade fever, chills, and asthenia, and 4 days before admission, he had constipation, malaise, generalized weakness, anorexia, nausea, and vomiting. On admission, the patient was afebrile and hypotensive, with a heart rate of 105 and an oxyhemoglobin saturation on room air of 95%. Hyponatremia, lymphopenia, elevated C-reactive protein, and ferritin were observed in complementary tests. Computed tomography (CT) scan showed findings consistent with COVID-19 bilateral bronchopneumonia, and an increase in size and blurring (loss of the Y shape) of both adrenals indicative of acute bilateral adrenal hemorrhage. The patient tested negative by reverse transcription polymerase chain reaction (RT-PCR) of nasopharyngeal swab, yet positive for IgG and IgM by ELISA, suggesting COVID-19 diagnosis.

## CASE REPORT

A 70-year-old Dominican Republic man living in Spain for more than 20 years with a medical history of mild psoriasis indicated having lower back pain for 10 days which did not improve with acetaminophen. Fifteen days before pain onset, he had low-grade fever, chills, and asthenia. Neither cough nor dyspnea was reported. Four days before admission, he had constipation, malaise, generalized weakness, anorexia, nausea, and vomiting.

**Figure 1.**
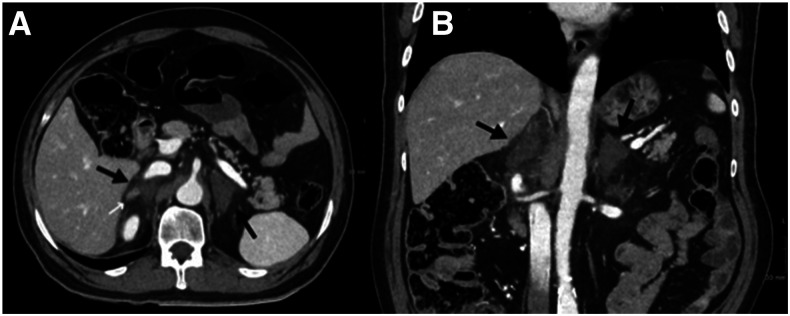
Axial (**A**) and coronal (**B**) images of abdominal CT scan with IV contrast. A marked increase in the dimensions of both adrenal glands with frayed edges, and with a general hypodense appearance (black arrows) is identified. A more attenuating pseudo-nodular image is identified within the right adrenal gland, which probably corresponds to undamaged tissue (white arrow). In addition, they are also associated with moderate inflammatory changes due to contiguity, with trabeculation of the peri-adrenal mesenteric fat. These findings are compatible with bilateral adrenal hematomas.

On admission, the patient was afebrile and hypotensive, with a heart rate of 105 and an oxyhemoglobin saturation on room air of 95%. On examination, a distended abdomen was noted. No bruises or signs of trauma were on the skin. Further investigation revealed hemoglobin and platelets within normal limits (WNLs), and elevated leukocytes (12,020 cells/μL [normal range 3,600–10,500 cells/μL]) with 80% (9,620 cells/μL) neutrophils, whereas the lymphocyte count (1,120 cells/μL) was WNLs (1,100–4,500 cells/μL). Sodium concentration was low (127 [136–145 mmol/L]) but with normal potassium level (4.5 [3.5–5.1] mmol/L); elevated liver enzymes (aspartate aminotransferase 227 IU/L [< 40], alanine transaminase 408 IU/L [< 35], and gamma-glutamyltransferase 129 IU/L [< 73]); elevated C-reactive protein concentration (137.7 mg/L [0.0–0.5]), cardiac troponin I (151.9 ng/L [< 53.5]), and ferritin (1,877 ng/mL [22–322]); and elevated D-dimer (960 ng/mL [0–500]). CT scan showed findings consistent with COVID-19 bilateral bronchopneumonia (CORADS 5 score, which implies a very high level of suspicion for pulmonary involvement by COVID-19 based on typical CT findings).^1^ Because an intestinal pseudo-obstruction was suspected, a CT scan was performed, showing small bowel dilatation and increase in size and blurring (loss of the Y shape) of both adrenals indicative of acute bilateral adrenal hemorrhage (BAH) (Figure 1).

The patient tested negative by RT-PCR of nasopharyngeal swab twice 48 hours apart, yet positive for IgG and IgM by ELISA (Vircell, Parque Tecnológico de la Salud, Granada, Spain), suggesting COVID-19 diagnosis. Other infections, specially imported ones (*Strongyloides*, HIV, syphilis, hepatitis B, hepatitis C, human T-lymphotropic virus, chlamydia, *parvovirus*, *cytomegalovirus*, and *Mycoplasma*), were ruled out.

Because of clinical and radiological suspicions of acute adrenal insufficiency, supportive fluid resuscitation and 300 mg/day of hydrocortisone by intravenous infusion were started, followed by oral therapy. COVID-19 was treated with off-label hydroxychloroquine for 5 days; heparin prophylaxis was initiated. The patient was discharged 9 days after clinical status, and blood analysis results improved as constipation resolved.

Any basal cortisol or stimulated cortisol could be performed in the acute phase because the patient was already under treatment with steroids. One month after discharge, the patient was on hydrocortisone 20 mg three times daily (10 mg/5 mg/5 mg), and after 16 hours without hydrocortisone supplementation, corticotrophin stimulation test with 250 µg adrenocorticotropic hormone was performed to confirm the diagnosis. Cortisol measurements were performed before, 30 and 60 minutes after IV administration. Our patient peak of cortisol at 30 and 60 minutes was less than 18 µg/dL, indicating adrenal insufficiency (basal: 2.1 mcg/dL, 30″: 2.89 µg/dL and 60″: 3.11 µg/dL).

At present, the patient is asymptomatic, he is on glucocorticoid replacement therapy with hydrocortisone, and no mineralocorticoid replacement is needed, with renin and aldosterone within normal ranges, blood pressure in range, and without ionic alterations.

## DISCUSSION

Adrenal hemorrhage is a rare condition, with unknown incidence, being estimated at an incidence of 0.14–1.8% of BAH based on postmortem studies. The importance of this entity relies on its potentially fatal consequences, with a mortality rate of 15% even after treatment.^2^ Traumatic BAH is extremely rare. The most frequent causes are surgery (including unrelated procedures), severe illness, trauma, severe sepsis (e.g., Waterhouse-Friedrichsen syndrome associated with meningococcal septicaemia), hypercoagulable states (including antiphospholipid antibody syndrome), burns, and anticoagulant treatment.^3^ Although adrenal hemorrhage is mostly described in the context of sepsis because of gland hypertrophy in response to infection, up-to-date this is not yet described in relation to COVID-19. As the pandemic is still in progression, we are not completely aware of all the coagulation disorders implicated. Preliminary reports on SARS-CoV-2 pandemic outcomes have shown that infected patients commonly develop coagulation disorders both disseminated intravascular coagulation (DIC) and thrombosis characterized by elevations in fibrinogen and D-dimer levels, in correlation with a parallel rise in markers of inflammation. Other markers of this disorder are a prolonged prothrombin time and a mild thrombocytopenia.^4^

Some patients infected by this novel virus are at risk of developing DIC with fulminant activation of coagulation and consumption of coagulation factors.^4^

The pathogenic mechanism implicated in BAH is still unknown. The key to understanding it resides in its vascular anatomy. Adrenal glands receive vascularization from three adrenal arteries from the aorta, the inferior phrenic artery, and the renal artery; this results in multiple arterioles forming a vascular plexus.^2,5^ However, the adrenal gland is drained exclusively by the central adrenal vein, producing a point that is more vulnerable in the event that there is venous vasoconstriction during shock, which can lead to an overload of these collecting capillaries. An excessive increase in blood pressure or a thrombotic event at the level of the adrenal vein could cause a bleed. In COVID-19, several determining factors could occur: an increase of catecholamines that predisposes to an increase in the adrenal flow,^6^ a shock that produces venous constriction, and a prothrombotic environment that would worsen the picture.^4,7^

Symptoms of a BAH are similar to those of COVID-19 (fever, asthenia, nausea, malaise, etc.), so they could go unnoticed. This is the first published case describing BAH in a COVID-19 patient. Failure to recognize and treat adrenal insufficiency can be fatal. We believe this comprehensive description of BAH in relation to COVID-19 will raise awareness and promote early diagnosis and treatment.
